# Enhancement of iron, zinc, and calcium bioaccessibility and bioavailability in green gram (*Vigna radiata* L.) supplemented with buttermilk through phytate reduction: an *in vitro* dietary evaluation

**DOI:** 10.3389/fnut.2026.1756171

**Published:** 2026-01-30

**Authors:** Bindhu Varshanath, Delvin T. Robin, Sudha Meera, Anusree Dileep, Shylaja Archana, Nidhin Chandran, Madhavan Vandana Rani

**Affiliations:** Department of Swasthavritta (Social and Preventive Medicine), Amrita School of Ayurveda, Amritapuri, Amrita Vishwa Vidyapeetham, Kollam, India

**Keywords:** Ayurveda, bioaccessibility, bioavailability, buttermilk, green gram, phytate, *takra*

## Abstract

**Introduction:**

Plants supply essential nutrients such as vitamins, minerals, proteins, and dietary fiber, which are required for human nutrition. However, in plant-based diets, specific compounds like phytates can inhibit the absorption of nutrients, leading to potential health problems. Green gram (*Vigna radiata L.*), a regularly used plant, contains phytic acid at levels ranging from 0.4 to 1.4%. The high levels of phytic acid in green gram can impede the absorption of essential minerals. Systematic alterations to dietary approaches are needed to resolve this issue. Ayurveda mentions the concept of adjuvant (*Anupāna*) to enhance the availability of nutrients and address any pitfalls in nutrient absorption. This research aims to ascertain if buttermilk (*Takra*) can serve as a nutritive enhancer in the diet and improve nutrient absorption from green gram.

**Methods:**

Bioaccessibility of iron, calcium, and zinc in four samples namely- uncooked green gram, cooked green gram, buttermilk and cooked green gram treated with butter milk was assessed using the INFOGEST digestion process. The digested samples were then analyzed by inductively coupled plasma mass spectrometer (ICP-MS) to determine the levels of these micronutrients. Moreover, the bioavailability of these micronutrients is evaluated by treating Caco-2 cells with the final digest and analyzing them using ICP-MS.

**Results:**

The statistical analysis exhibits that the final digest of cooked green gram treated with buttermilk had considerably higher amounts of bio accessible and bioavailable iron, calcium, and zinc than the untreated sample. Improved bio accessibility and bioavailability were underlined by a significant rise of 68% in iron, 57% in calcium, and 23% in zinc in the buttermilk treated samples.

**Conclusion:**

This substantiates Ayurvedic principles by demonstrating that the specific adjuvant, buttermilk increases the bio accessibility and bioavailability of micronutrients such as iron, calcium, and zinc in green gram.

## 1 Introduction

Foods obtained from plants are considered extremely nutritious for their abundance of essential minerals, vitamins, proteins, and dietary fiber, which are all key components of a healthy diet. The persistent deficiency of protein, energy and micronutrients detrimentally affects future cognitive and economic productivity. Utilizing food as a source of various nutrients is a significant approach to combat malnutrition. Nevertheless, the presence of antinutritional compounds in plant-based diets poses a challenge in attaining optimal dietary outcomes. These compounds, such as phytates, prevent the absorption of vital nutrients essential for overall health (1). Green gram, scientifically known as *Vigna radiata L.* is the most widely used legume, due to its high protein and mineral content. Compositional analyses have shown it contains substantial amounts of macronutrients and essential minerals, including protein (~228–278 g kg^−1^), calcium (~166–341 mg kg^−1^), iron (~40–349 mg kg^−1^), and zinc (~27–34 mg kg^−1^) along with other minerals such as potassium and magnesium, which contribute to its nutritional value in human diets (2). However, the presence of antinutritional factors such as phytic acid, tannins, and phenolic compounds in green gram can chelate divalent mineral ions, reducing the fraction of minerals that are soluble and absorbable under gastrointestinal conditions (3). Buttermilk also contains water-soluble components like milk protein, lactose, and minerals (4). The ratios between phytate and minerals in green gram products surpasses critical thresholds, potentially inhibiting optimal mineral absorption (1). For example, the ratios for phytate/iron, phytate/calcium, and phytate/zinc (5.9 for phytate/iron, 0.13 for phytate/calcium, and 11.5 for phytate/zinc) are above the threshold values (2.48 for phytate/iron, 0.05 for phytate/calcium, and 1.96 for phytate/zinc) (5, 6). This highlights the need for descriptive changes in meals to alleviate the impeding effects of phytates on nutrient uptake. Bio accessibility is the fraction of a nutrient released from the food matrix during gastrointestinal digestion, available for intestinal uptake and bioavailability is the proportion of an ingested nutrient that is absorbed and available for physiological use or storage (7). Conventional food and nutrition supplementation programs must address challenges of bioavailability and malabsorption to manage micronutrient deficiency optimally. In developing nations, food fortification has proven effective in reducing micronutrient deficiencies, but due to diverse eating habits and restricted access to fortified foods, alternative methods of supplementation should be explored, preferably through established dietary strategies.

In *Āyurveda*, food is considered as the fundamental entity for maintaining health. Ayurveda utilizes the concept of adjuvant (*Anupāna*) to enhance the availability of nutrients and address any difficulties in nutrient absorption. The term “*Anupāna*” describes the deliberate use of vehicles or carriers, like beverages or particular foods, to optimize the effectiveness and absorption of ingested materials. *Acharya Vāgbhata* advocates the use of buttermilk (*takra*), as an adjuvant, owing to its potential to enhance the nutritional value of cooked green gram (*Vigna radiata L.*) (8). The present study targets to assess the effect of buttermilk as adjuvant to green gram by assessing the bio accessibility and bioavailability of iron, calcium, zinc from green gram when consumed without and with adjuvant.

## 2 Materials and methods

The study was conducted in the Athmic Biotech solutions Pvt. ltd R&D lab, Thiruvananthapuram, and Amrita Centre for Advanced Research in *Āyurveda*, Amrita Vishwa Vidyapeetham, Kollam, Kerala.

The study utilized four samples, namely uncooked green gram, cooked green gram, buttermilk and cooked green gram treated with buttermilk. The green gram samples were sourced from the Onattukara Regional Agricultural Research Station, Kerala Agricultural University, India. Analytical-grade chemicals, double-distilled water, and meticulous equipment cleaning were employed throughout the investigation, with the INFOGEST in-vitro digestion process incorporating enzymes from Nice Chemicals Private Limited. The digestion was followed by centrifugation at 400 rpm, and acid digestion for ICP-MS analysis was performed using the ANTON-PAR Multiwave GO Microwave digestive device.

### 2.1 Pre-treatments procedures

#### 2.1.1 Authentication of green gram

The procured green gram (*Vigna radiata* L.) seeds incorporated into herbarium sheet, representing a desiccated plant specimen affixed onto a paper sheet, which has been duly authenticated and endorsed by botanical experts (9).

#### 2.1.2 Preparation of raw green gram samples

Raw green gram seeds were manually sorted to remove broken, shrivelled, discolored seeds, and extraneous matter. The cleaned seeds were sun dried and stored in airtight glass containers at room temperature (27 ± 10 °C) for around 4 weeks, following standard pulse sample preparation procedures (10).

#### 2.1.3 Preparation of cooked green gram

One hundred green gram seeds were soaked in distilled water for 7 h. After soaking, the water was removed, measured, and filtered through ashless filter paper (11). The soaked grains, along with the required volume of cooking water, were transferred into heat-resistant glass jars. For every 100 g of soaked grains, 50 mL of distilled water was added as the cooking medium, which was retained after cooking to facilitate homogenization. The glass jars were placed inside a standard pressure cooker, and pressure cooking was carried out for 10 min at maximum power. The cooked samples were then homogenized, refrigerated until further analysis, and 500 mg of the finely ground sample was used for bio accessibility and bioavailability assessment (12).

#### 2.1.4 Preparation of buttermilk

Fresh cow’s milk was procured from an adjacent hamlet, where the cow was fed exclusively on grass. The milk was thoroughly boiled over a medium flame, cooled and kept overnight at ambient temperature. On the following morning, the curd was separated from the whey. A measured volume (100 mL) of curd was churned with 25 mL of distilled water until complete separation of fat occurred, yielding buttermilk following traditional dairy preparation methods (13).

#### 2.1.5 Preparation of green gram–buttermilk blend

**Figure 1:**
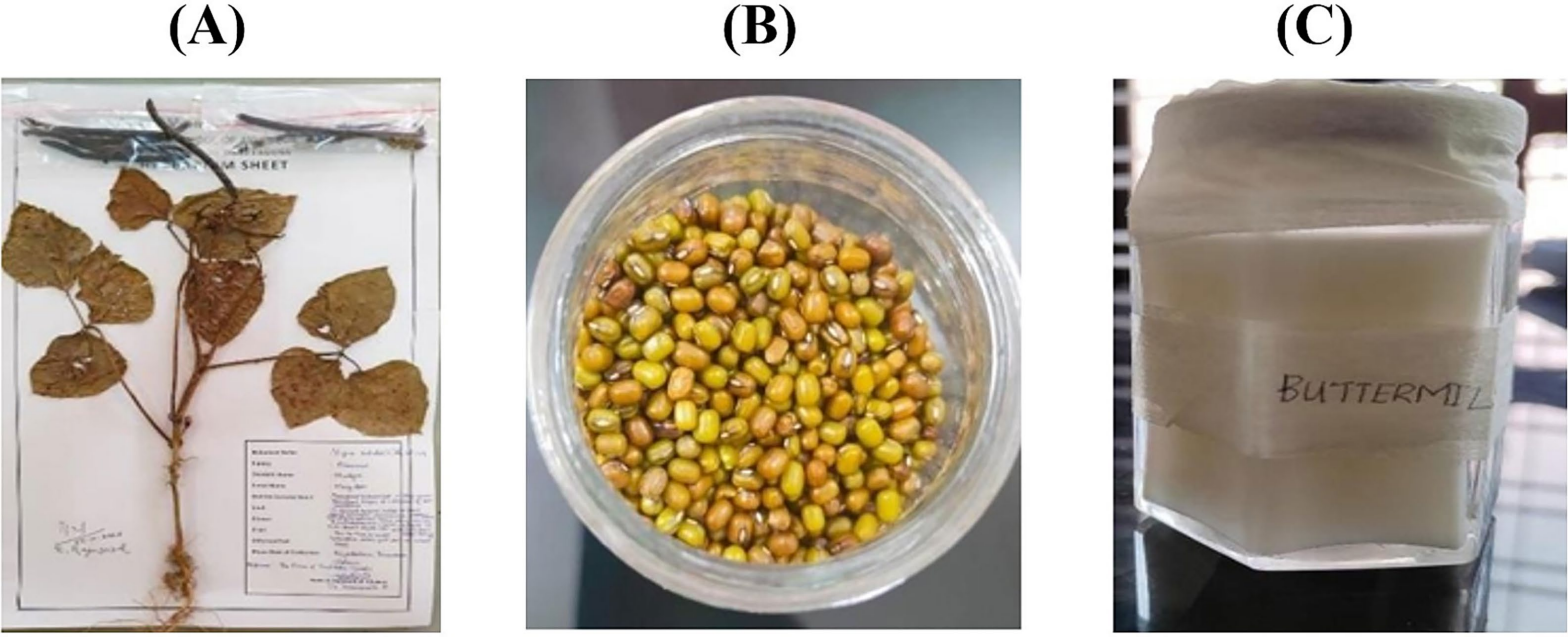
Results of pre-treatments procedures **(A–C)**. **(A)** Herbarium sheet of green gram (*Vigna radiata* L.) authenticated by botanical experts; **(B)** Raw green gram sample used for preparation; and **(C)** Freshly prepared buttermilk, utilized in combination for bioavailability and bio accessibility experiments.

Cooked green gram homogenate was blended with freshly prepared buttermilk in a standardized ratio of 1:1 (w/v), homogenized to obtain a uniform mixture, and immediately subjected to *in vitro* digestion and mineral bio accessibility analysis (14). Results of Pre-treatment procedures are shown in Figure 1.

### 2.2 Estimation of iron, calcium, zinc content in unprocessed green gram and buttermilk

Samples of uncooked green gram and freshly prepared buttermilk were subjected to cold acid digestion using a nitric acid–hydrogen peroxide mixture, following standard AOAC guidelines for mineral analysis. 0.5 g of sample was digested with 5.0 mL concentrated nitric acid (HNO₃; 65% w/w) and 2.0 mL hydrogen peroxide (H₂O₂; 30% w/w) at 180 °C for 1 h in an ANTON-PAR Multiwave GO Microwave digestion system until a clear solution was obtained. After cooling, the digests were filtered, transferred to 50 mL volumetric flasks, and diluted to volume with ultrapure water. The concentrations of iron (Fe), calcium (Ca), and zinc (Zn) were determined using inductively coupled plasma mass spectrometry (ICP-MS) under manufacturer-recommended standard plasma operating conditions (15).

### 2.3 Determination of phytase activity of buttermilk using hydrolysis of phytic acid method

#### 2.3.1 Method

Phytase activity was determined based on the quantitative estimation of inorganic phosphate released from sodium phytate following enzymatic hydrolysis. The assay was conducted under controlled conditions of pH 5.5 and 37 °C temperature, and enzyme activity was expressed in activity units (U), as commonly adopted for phytase characterization and enzymology studies (16–19). Potassium dihydrogen phosphate (KH₂PO₄) was used to generate a standard curve for inorganic phosphate quantification.

#### 2.3.2 Inorganic phosphate standard curve

A stock solution of KH₂PO₄ was prepared by dissolving 0.6804 g of pre-dried KH₂PO₄ in distilled water and making up the volume to 100 mL. This solution was serially diluted to generate a standard curve for inorganic phosphate estimation, following established phosphate determination protocols (18, 19). Aliquots of 1.0, 1.5, 2.0, 2.5, 3.0, and 4.0 mL were transferred into centrifuge tubes, and the final volume was adjusted to 4.0 mL using 0.22 mol·L^−1^sodium acetate buffer (pH 5.5 ± 0.01).

#### 2.3.3 Reagents

A 0.5 mol·L^−1^ trichloroacetic acid (TCA) solution was prepared in distilled water. The substrate solution consisted of 200 mmol·L^−1^sodium acetate buffer (pH 5.5) containing 0.1% (w/v) sodium phytate. Freshly each day, 5.0 g of ferrous sulfate heptahydrate (FeSO₄·7H₂O) was dissolved in 90 mL of distilled water, to which 10.0 mL of 8.0% (w/v) ammonium molybdate solution was added. Ammonium molybdate (8.0 g) was initially dissolved in 50 mL of distilled water, and the volume was then adjusted to 100 mL to obtain an 8.0% (w/v) solution. Subsequently, 27.0 mL of 10 mol·L^−1^ sulfuric acid (H₂SO₄) was added, and the solution volume was adjusted to 100 mL with distilled water. These reagents were prepared following standard protocols for phosphate determination in phytase assays (17–19).

#### 2.3.4 Phytase assay

The substrate was prepared using 200 mmol·L^−1^ sodium acetate buffer (pH 5.5) containing 0.1% (w/v) sodium phytate and was freshly prepared and stored at 4 °C for no longer than 24 h prior to analysis. The assay reaction mixture consisted of 0.5 mL of substrate solution and 0.5 mL of phytase enzyme solution. All assay components were pre-equilibrated to 50 °C, and the reaction mixture was incubated at 50 °C for 15 min, consistent with optimal phytase assay conditions reported in earlier studies (16, 18, 19). The reaction was terminated by the addition of 1.0 mL of TCA solution.

All assays were performed in duplicate along with appropriate blanks. For the blank, substrate and enzyme solutions were incubated separately at 50 °C for 15 min, followed by the addition of TCA to the enzyme prior to mixing with the substrate. After termination, 1.0 mL of reagent A was added to all tubes (assay and blank), and absorbance was measured at 660 nm after standing for 5 min at room temperature.

#### 2.3.5 Calculation of phytase enzymatic activity

One unit (1 U) of phytase activity was defined as the amount of enzyme that catalyzes the release of 1 μmol of inorganic phosphate per minute under the specified assay conditions (18, 19). The quantity of inorganic phosphate released was calculated from the KH₂PO₄ standard curve and expressed in nanomoles. Since 0.5 mL of enzyme solution was used in the assay, the measured activity was multiplied by 2 to express phytase activity as units per milliliter (U·mL^−1^) of enzyme preparation.

### 2.4 Estimation of phytate in uncooked, cooked green gram and cooked green gram with buttermilk using spectrophotometry method

Phytate content was estimated using the spectrophotometric method described by Wheeler and Ferrel (20), which is widely employed for the quantitative determination of phytic acid in plant-based foods (21–23). 5 g of finely ground sample was weighed into a 125 mL conical flask and extracted with 50 mL of 3% (w/v) trichloroacetic acid (TCA) by mechanical shaking for 3 h at room temperature. The extract was centrifuged at 3,000 rpm for 5 min, and 10.0 mL of the clear supernatant was transferred to a 40 mL centrifuge tube. To this, 4.0 mL of ferric chloride solution (containing 2 mg Fe^3+^·mL^−1^ in 3% TCA) was added. The mixture was heated in a boiling water bath for 45 min, cooled to room temperature, and centrifuged for 10–15 min. After adding 1–2 drops of 3% (w/v) sodium sulfate (Na₂SO₄) in 3% TCA, the supernatant was carefully decanted. The precipitate was washed by dispersing it in 25 mL of 3% TCA, reheated in a boiling water bath for 10–15 min, and centrifuged again. The washed precipitate was then repeatedly rinsed with distilled water and subsequently dissolved in a small volume of distilled water containing 3.0 mL of 1.5 mol·L^−1^ sodium hydroxide (NaOH). The volume was adjusted to approximately 30 mL, followed by heating in a boiling water bath for 30 min. The hot solution was filtered through Whatman No. 2 filter paper, and the residue was washed thoroughly with 60–70 mL of hot distilled water. The filter paper containing the precipitate was dissolved using 40 mL of hot 3.2 mol·L^−1^ nitric acid (HNO₃) and quantitatively transferred to a 100 mL volumetric flask. The filter paper was rinsed with hot distilled water, and the washings were combined in the same flask and made up to volume with distilled water. From this solution, a 0.5 mL aliquot was transferred to a 10 mL volumetric flask, followed by the addition of 2.0 mL of 1.5 mol·L^−1^ potassium thiocyanate (KSCN). The volume was adjusted with distilled water, and absorbance was measured immediately (within 1 min) at 480 nm using a UV–visible spectrophotometer.

**Figure 2:**
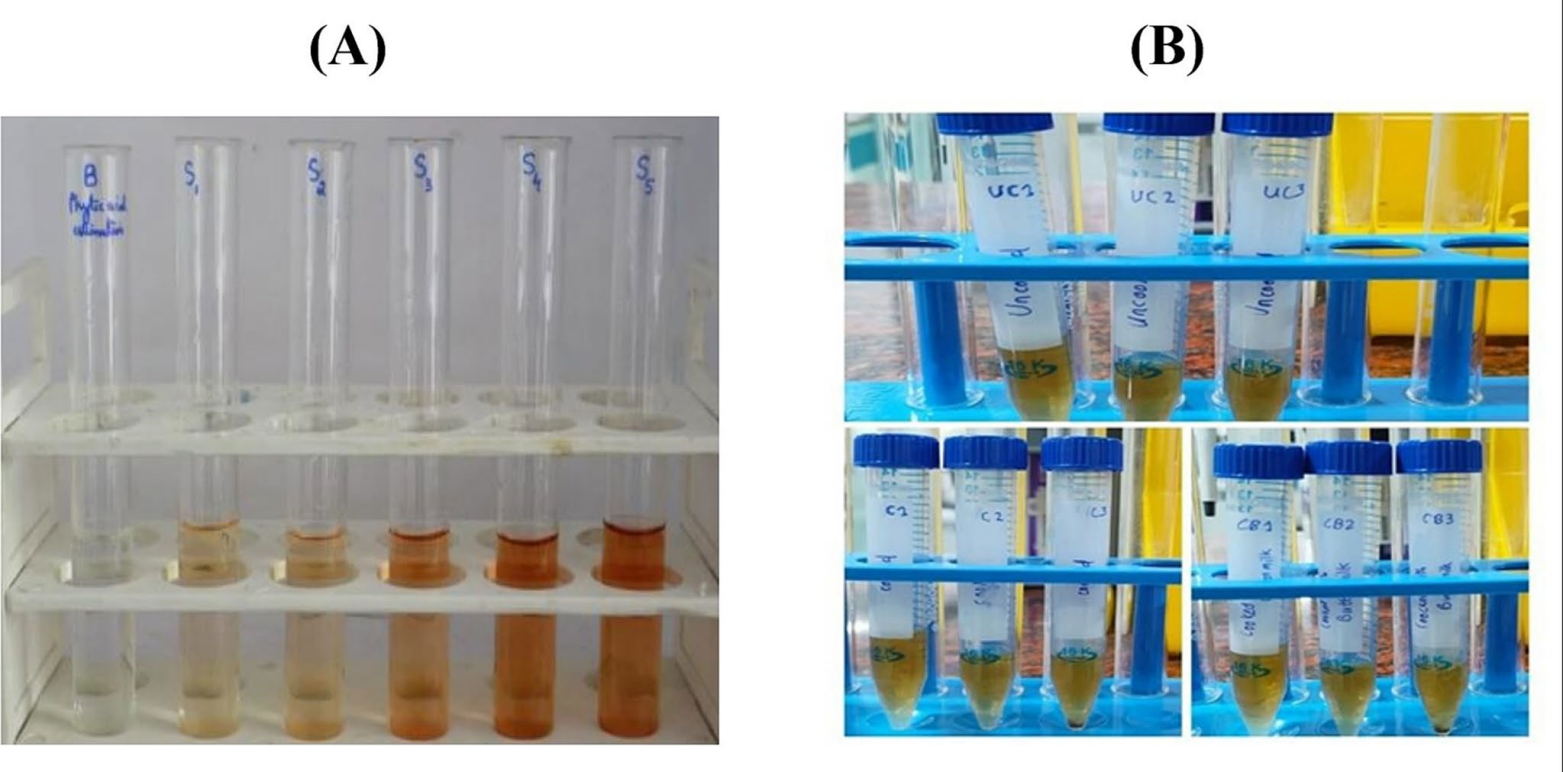
Spectrophotometric estimation of phytate content as per Wheeler and Ferrel method. **(A)** Phytic acid blank estimation for calibration, and **(B)** Phytate quantification in uncooked, cooked green gram, and cooked green gram with buttermilk recorded at 480 nm.

Ferric ion concentration was determined using a standard calibration curve prepared with ferric nitrate [Fe(NO₃)₃] solutions. Phytate phosphorus content was calculated from iron concentration using a molar ratio of iron to phosphorus of 4:6, as described in the original method (20) and subsequent applications (21–23). The spectrophotometric estimation of phytate content using the Wheeler and Ferrel method is illustrated in Figure 2.


Phytate(g/100g)=(6/4×A×C×20×10×50×100)/1000×S

Where: A = optical density = concentration corresponding to optical density, S = weight of sample (0.1 g).

### 2.5 Methodology of bio accessibility of the micronutrient iron, calcium and zinc using *in vitro* simulated digestion using INFOGEST protocol

**Figure 3:**
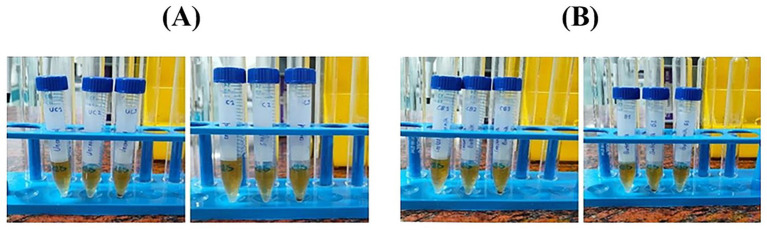
Estimation of bio accessibility of iron, calcium, and zinc using the *in vitro* simulated digestion (INFOGEST) protocol. **(A)** Uncooked and cooked green gram (digested) **(B)** cooked green gram treated with buttermilk and buttermilk (digested).

The INFOGEST Protocol was used to perform simulated digestion on each sample. Salivary, gastric, and intestinal fluids were prepared and adjusted to specific pH levels. Enzyme activities and bovine bile salt content were assessed (14). The oral phase involved mixing liquid food with simulated salivary fluid and adjusting to pH 7. The gastric phase involved mixing the oral bolus with simulated gastric fluid and adjusting to pH 3.0. The intestinal phase involved mixing gastric chyme with simulated intestinal fluid and adjusting to pH 7. After digestion, samples were centrifuged and extracted for iron, calcium, and zinc quantification (15). Figure 3 shows the estimation of bio accessibility of iron, calcium, and zinc using the *in vitro* simulated digestion (INFOGEST) protocol.

### 2.6 Methodology of bioavailability of the micronutrient iron, calcium and zinc using Caco-2 cell analysis

#### 2.6.1 Cell culture media and maintenance

Cells were cultured in Dulbecco’s Modified Eagles Medium (DMEM-HiMedia) supplemented with 10% heat-inactivated fetal bovine serum (FBS) and a 1% antibiotic cocktail containing Penicillin (100 U·mL^−1^), Streptomycin (100 μg·mL^−1^), and Amphotericin B (2.5 μg·mL^−1^). The cell-containing tissue culture flasks (75 cm^2^) were incubated at 37 °C in a 5% CO_2_ environment with humidity using a Galaxy® 170 Eppendorf cell culture incubator from Germany (24).

#### 2.6.2 Cell line preparation

Cells (3.0 × 10^5^ cells·well^−1^) were seeded on six-well plates and acclimatized for 24 h at 37 °C in a 5% CO_2_ atmosphere. Digested samples of cooked and cooked green gram with buttermilk were filtered through a 0.2 μm Millipore syringe filter, were added to six-well sterile microtiter plates at concentrations of 6.25, 12.5, 25, 50, and 100 μg·mL^−1^ in DMEM medium. Untreated cells served as controls (24).

#### 2.6.3 Quantification

**Figure 4:**
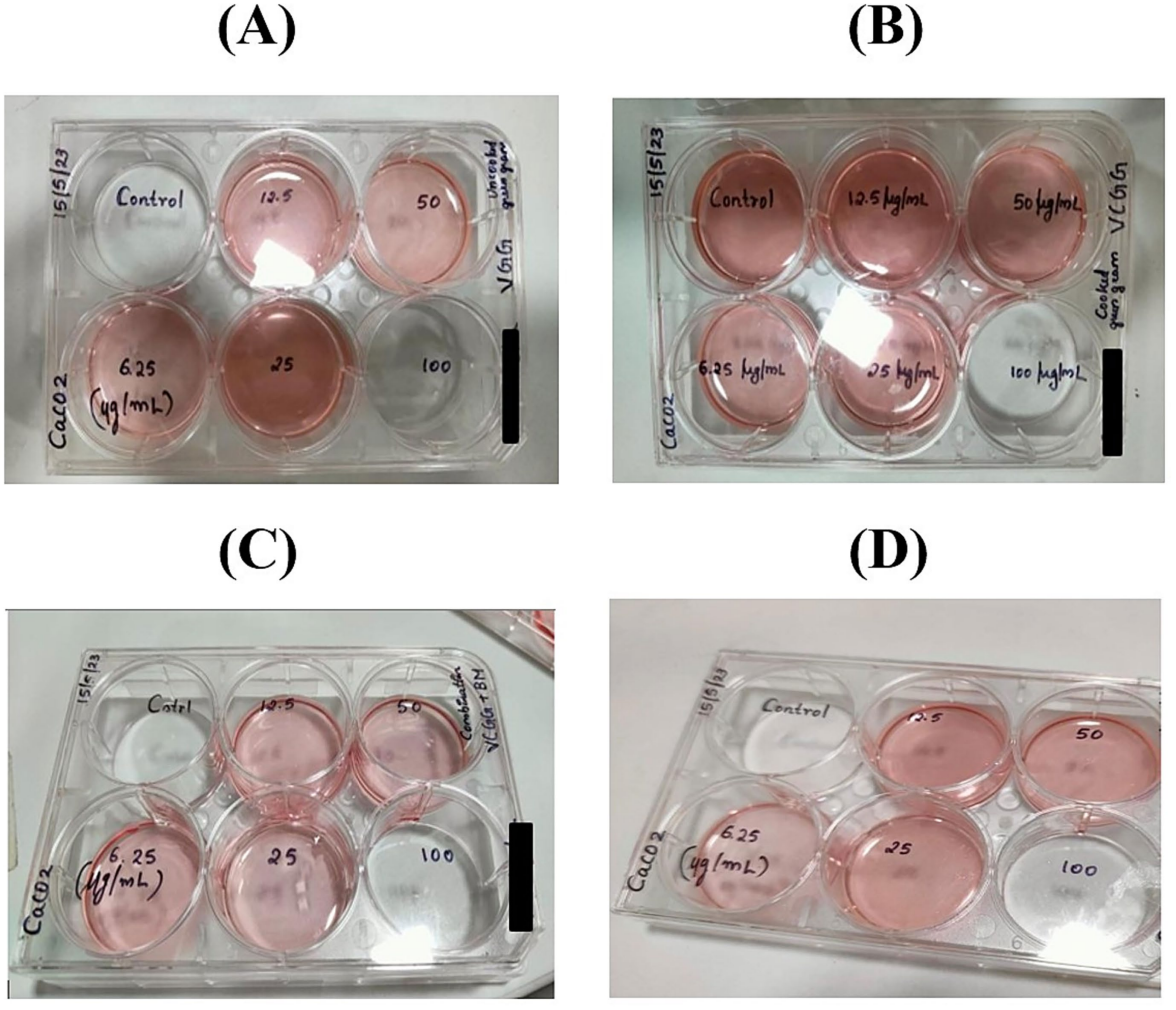
Bioavailability estimation of micronutrients (iron, calcium, and zinc) using the Caco-2 cell model. **(A)** Uncooked green gram, **(B)** cooked green gram, **(C)** cooked green gram treated with buttermilk, and **(D)** buttermilk.

After a 24-h incubation, ICP-MS analysis was conducted. For quantifying bio accessible and bioavailable iron, calcium, and zinc, the cell-treated final digested samples were subjected to hydrogen peroxide and nitric acid digestion at 180 °C for 1 h, followed by ICP-MS analysis to determine bioavailable levels, adhering to recommended guidelines (15). Figure 4 shows bioavailability estimation of micronutrients using Caco-2 cell model.

### 2.7 Statistical analysis

All determinants were carried out in triplicates, and the average value was taken. Statistical analysis of data was done by applying one way ANOVA test in phytic acid determination and Mann–Whitney U test for the determination of bio accessible and bioavailable content of iron, calcium and zinc. The differences were considered statistically significant of *p* value<0.05.

## 3 Results

### 3.1 Estimation of iron, calcium, zinc in raw green gram and buttermilk

In raw green gram, calcium was present at the highest concentration (1092.84 ± 0.22 mg·kg^−1^), followed by iron (59.92 ± 0.14 mg·kg^−1^) and zinc (29.71 ± 0.06 mg·kg^−1^). In buttermilk, calcium again appeared as the predominant micronutrient (124.19 ± 0.40 mg·kg^−1^), followed by zinc (23.52 ± 0.09 mg·kg^−1^) and iron (5.36 ± 0.04 mg·kg^−1^). The low standard deviation observed across triplicate determinations indicates minimal analytical variability and good precision. All measured concentrations were well above the detection limit of 0.05 mg·kg^−1^ (25), confirming the reliability of mineral estimation in both raw green gram and buttermilk. The concentrations of iron, calcium, and zinc in raw green gram and buttermilk are presented in Table 1.

**Table 1:** Results of estimated iron, calcium, zinc in raw green gram and buttermilk.

Sl. No.	Sample	Parameter	Mean ± SD (mg kg^−1^)
1	Uncooked green gram	Iron (Fe)	59.92 ± 0.14
2		Calcium (Ca)	1092.84 ± 0.22
3		Zinc (Zn)	29.71 ± 0.06
4	Buttermilk	Iron (Fe)	5.36 ± 0.04
5		Calcium (Ca)	124.19 ± 0.40
6		Zinc (Zn)	23.52 ± 0.09

Values are expressed as mean ± standard deviation (SD) of triplicate determinations (*n* = 3). The detection limit for iron, calcium, and zinc was 0.05 mg kg^−1^. Analysis was performed using inductively coupled plasma–mass spectrometry (ICP-MS).

### 3.2 Determination of phytase activity of buttermilk using hydrolysis of phytic acid method

The release rate of organic phosphate in buttermilk was 1.47 μmol·min^−1^, and the enzyme activity was 2.93 U·mL^−1^. This indicates good enzymatic activity, demonstrating rapid liberation of phosphate under the test conditions (26).

### 3.3 Estimation of phytate in uncooked green gram, cooked green gram, cooked green gram with buttermilk using spectrophotometry method

Phytic acid content was highest in uncooked green gram (53.08 ± 0.03 μg·mL^−1^; 1.58 ± 0.02 g·100 g^−1^). Cooking resulted in a reduction in phytic acid levels to 46.42 ± 0.36 μg·mL^−1^, corresponding to 1.20 ± 0.02 g·100 g^−1^. A further and pronounced decrease was observed in cooked green gram treated with buttermilk, where phytic acid content declined to 32.38 ± 0.04 μg·mL^−1^, equivalent to 0.54 ± 0.01 g·100 g^−1^. The low standard deviation across triplicate determinations indicates minimal analytical variability and good precision. The phytic acid content of uncooked, cooked, and buttermilk-treated cooked green gram is presented in Table 2.

**Table 2:** Results of estimation of phytic acid.

Sl. No	Sample	Phytic acid (μg mL^−1^)	Phytic acid (g 100 g^−1^)
1	Uncooked green gram	53.08 ± 0.03	1.58 ± 0.02
2	Cooked green gram	46.42 ± 0.36	1.20 ± 0.02
3	Cooked green gram with buttermilk	32.38 ± 0.04	0.54 ± 0.01

Values are expressed as mean ± standard deviation (SD) of triplicate determinations (*n* = 3). Phytic acid concentration was estimated spectrophotometrically at 470 nm.

### 3.4 Estimation of bio accessibility of iron, calcium, zinc in the samples using the INFOGEST digestion protocol followed by ICP-MS analysis

In uncooked green gram, calcium was present at the highest concentration (523.47 ± 0.60 mg·kg^−1^), followed by zinc (12.49 ± 0.06 mg·kg^−1^) and iron (10.45 ± 0.39 mg·kg^−1^). After digestion of cooked green gram, the concentrations of iron (Fe), calcium (Ca), and zinc (Zn) were 12.50 ± 0.06 mg·kg^−1^, 387.62 ± 0.23 mg·kg^−1^, and 18.19 ± 0.14 mg·kg^−1^, respectively. In digested buttermilk, calcium remained the most abundant mineral (92.25 ± 0.20 mg·kg^−1^), followed by zinc (6.73 ± 0.15 mg·kg^−1^) and iron (3.63 ± 0.02 mg·kg^−1^). When cooked green gram was digested in the presence of buttermilk, the mineral levels increased substantially, with iron at 27.64 ± 0.45 mg·kg^−1^, calcium at 749.98 ± 0.32 mg·kg^−1^, and zinc at 22.72 ± 0.03 mg·kg^−1^, all well above the detection limit of 0.05 mg·kg^−1^ (25). The close agreement among triplicate determinations demonstrates high analytical precision and reproducibility of both the INFOGEST digestion protocol and the ICP-MS quantification. Compared with uncooked green gram, cooked green gram, and buttermilk alone, the buttermilk-treated sample showed a substantial increase in Fe, Ca, and Zn concentrations, indicating that the presence of buttermilk significantly enhanced micronutrient bio accessibility.

### 3.5 Estimation of bioavailability of iron, calcium, zinc in Caco-2 cells on treatment with the final digest of the samples followed by ICP-MS analysis

The estimated bioavailable levels of iron (Fe), calcium (Ca), and zinc (Zn) in the Caco-2 cell–treated digest of uncooked green gram were 7.09 ± 0.41 mg·kg^−1^, 592.89 ± 0.15 mg·kg^−1^, and 19.39 ± 0.01 mg·kg^−1^, respectively. Similarly, the cooked green gram digest showed bioavailable Fe, Ca, and Zn levels of 8.30 ± 0.02 mg·kg^−1^, 300.31 ± 0.27 mg·kg^−1^, and 17.26 ± 0.06 mg·kg^−1^, respectively. In buttermilk, the estimated bioavailable concentrations of Fe, Ca, and Zn were 2.78 ± 0.12 mg·kg^−1^, 76.90 ± 0.01 mg·kg^−1^, and 3.94 ± 0.06 mg·kg^−1^, respectively. In the cooked green gram digest treated with buttermilk, substantially higher bioavailable levels were recorded, with Fe at 25.78 ± 0.33 mg·kg^−1^, Ca at 698.68 ± 0.29 mg·kg^−1^, and Zn at 21.32 ± 0.49 mg·kg^−1^, respectively. Calcium consistently appeared in the highest concentration among the analysed micronutrients, whereas iron and zinc were present in lower but stable amounts. Compared with all other samples, the cooked green gram digest treated with buttermilk demonstrated a substantial enhancement in the bioavailability of all three micronutrients.

### 3.6 Estimation of phytate in uncooked green gram, cooked green gram and cooked green gram with buttermilk using spectrophotometry method

A one-way ANOVA test was performed. The difference was significant at the 0.05 level of significance. The concentration of phytate in green gram decreased after cooking and decreased even further after treatment with buttermilk. This reduction is evident in Table 3 when comparing the mean phytate concentration in buttermilk-treated cooked green gram (32.38 μg·mL^−1^) with uncooked (53.08 μg·mL^−1^) and cooked (46.42 μg·mL^−1^) green gram. The F-statistic was calculated to be 1579.33, which is much greater than the critical *F* value for *α* = 0.05 with 2 and 6 degrees of freedom. This indicates that buttermilk has a significant effect on the dephytinization of green gram when used as an adjuvant.

**Table 3:** Statistical analysis of estimation of phytate.

Sample	Mean	Sum of Squares (SS)	Error Sum of Squares (SSE)	F statistic
Uncooked green gram	53.08	245.66	0.03	1579.33
Cooked green gram	46.42	18.69	1.17	
Cooked green gram + buttermilk	32.38	398.96	0.07	
Total	43.96	663.31	1.27	

Mean Square Treatment (MST): 331.66, Mean Square Error (MSE): 0.20.

Statistical analysis was performed using one-way ANOVA.

### 3.7 Statistical analysis of bio accessible and bioavailable iron, calcium, zinc in cooked green gram with and without buttermilk

The bio accessibility of iron was significantly higher in cooked green gram treated with buttermilk (27.64 ± 0.450) compared to cooked green gram without buttermilk (12.50 ± 0.061). Similarly, the bioavailability of iron was significantly enhanced in the buttermilk-treated sample (25.78 ± 0.325) compared to the untreated cooked green gram (8.297 ± 0.015).

A significant improvement was also observed for calcium. The bio accessibility of calcium in cooked green gram treated with buttermilk (750.0 ± 0.323) was markedly higher than that in cooked green gram without buttermilk (387.62 ± 0.229). Likewise, calcium bioavailability was significantly greater in the buttermilk-treated sample (698.7 ± 0.289) compared to the sample without buttermilk (300.3 ± 0.278).

For zinc, the bio accessibility in cooked green gram treated with buttermilk (22.72 ± 0.026) was significantly higher than that observed in cooked green gram without buttermilk (18.19 ± 0.135). In addition, the bioavailability of zinc was significantly increased in the buttermilk-treated sample (21.32 ± 0.497) compared to the untreated cooked green gram (17.26 ± 0.057). Statistical analysis revealed that the *p*-values for iron, calcium, and zinc were less than the significance level of 0.05, indicating a statistically significant difference in both bio accessibility and bioavailability of these minerals between cooked green gram treated with and without buttermilk. The statistical analysis of bio accessible and bioavailable iron, calcium, and zinc in cooked green gram with and without buttermilk is presented in Table 4.

**Table 4:** Bio accessible and bioavailable iron, calcium, zinc in cooked green gram with and without buttermilk.

Mineral	Parameter	Sample	Mean ± SD	Mean Rank	*p*-value
Iron (Fe)	Bio accessibility	Without buttermilk	12.50 ± 0.061	2	0.049*
		With buttermilk	27.64 ± 0.450	5	
	Bioavailability	Without buttermilk	8.30 ± 0.015	2	0.049*
		With buttermilk	25.78 ± 0.325	5	
Calcium (Ca)	Bio accessibility	Without buttermilk	387.62 ± 0.229	2	0.049*
		With buttermilk	750.00 ± 0.323	5	
	Bioavailability	Without buttermilk	300.30 ± 0.278	2	0.049*
		With buttermilk	698.70 ± 0.289	5	
Zinc (Zn)	Bio accessibility	Without buttermilk	18.19 ± 0.135	2	0.049*
		With buttermilk	22.72 ± 0.026	5	
	Bioavailability	Without buttermilk	17.26 ± 0.057	2	0.046*
		With buttermilk	21.32 ± 0.497	5	

Data expressed as Mean ± Standard Deviation (SD).

^*^p < 0.05 considered statistically significant.

## 4 Discussion

### 4.1 Probable mode of action in the reduction of phytate in cooked green gram sample

The reduction of phytic acid in cooked green gram can be attributed to various mechanisms. Soaking increases water uptake and facilitate the leaching of water-soluble phytates into the soaking medium and increases the susceptibility of phytic acid to breakdown during subsequent cooking and enzymatic action (27). The application of heat during cooking further reduces phytic acid content, as boiling or prolonged heat treatments significantly decrease the concentration of phytic acid in legumes, likely by destabilizing heat-labile phytates and promoting hydrolysis. Cooking can also activate endogenous phytase enzymes, which catalyze the hydrolysis of phytic acid (1). pH changes during cooking and soaking processes may influence phytic acid stability and phytase activity, as acidic or variable conditions can enhance phytate breakdown. Furthermore, some of the phytic acid may leach into the cooking water, leading to a reduction in its content. Cooking induced cellular structure of green gram can expose phytic acid molecules to enzymatic action, thereby accelerating their degradation (28).

### 4.2 Probable mode of action in the reduction of phytate in buttermilk treated green gram sample

These include lowering of pH during fermentation, which promotes phytic acid degradation by enhancing mineral solubility and facilitating the action of endogenous or microbial phytases where present; the chelating properties of buttermilk that may compete with phytic acid for mineral binding sites; fermentation-induced structural changes in the green gram matrix; and synergistic interactions between buttermilk and green gram that collectively enhance the dephytinization process (23).

Together these mechanisms account for the significant impact of buttermilk as an adjuvant in promoting the dephytinization of green gram. The findings are consistent with Gupta et al., indicating that buttermilk, with its phytase enzymatic activity, has the potential to decrease anti nutrient content of rabadi (29), specifically phytic acid. This reduction is evidenced by the release of inorganic phosphate molecules, making minerals more bioavailable from the strong chelating complexes of phytate molecules and thereby potentially enhancing the nutritional quality of the dairy product.

### 4.3 Bio accessibility and bioavailability of the micronutrients

The present study was designed to scientifically validate the Ayurvedic concept of *anupāna* by examining the influence of buttermilk on the bio accessibility and bioavailability of iron, calcium, and zinc from green gram using the standardized INFOGEST *in vitro* digestion model followed by Caco-2 cell uptake assessment (30). The findings demonstrate that uncooked green gram exhibited higher initial mineral content compared to buttermilk alone; however, simulated gastrointestinal digestion resulted in a reduction in mineral bio accessibility, likely due to the formation of insoluble complexes and the presence of antinutritional factors such as phytic acid, which are known to limit mineral solubility during digestion. The incorporation of buttermilk during digestion markedly enhanced the bio accessibility of iron, calcium, and zinc compared to cooked green gram without buttermilk, indicating a positive modulatory effect of the adjuvant matrix on mineral release.

The enhanced iron bio accessibility observed in the presence of buttermilk may be attributed to its acidic nature and fermentation-derived organic acids, which are known to improve iron solubility by 55.36% and facilitate the reduction of ferric to ferrous iron, thereby enhancing non-heme iron availability during digestion (31). Similarly, the substantial increase in calcium bio accessibility by 48% may be explained by the formation of soluble calcium–organic acid complexes, particularly with lactic acid, which have been shown to improve calcium solubility and intestinal uptake in fermented dairy matrices (32). Although zinc bio accessibility increased by 19%, a lesser extent compared to iron and calcium, this trend is consistent with earlier reports indicating that zinc bio accessibility is strongly influenced by the presence of chelating compounds and antinutritional factors; fermentation and probiotic activity in buttermilk may partially mitigate these inhibitory effects by degrading zinc-binding compounds (33).

The higher bioavailability of iron (68%), calcium (57%), and zinc (23%) in buttermilk-treated digests, as evidenced by the Caco-2 cell model, suggests that buttermilk not only enhances mineral solubility but also promotes cellular uptake. Fermented dairy products have been reported to improve iron bioavailability through microbial metabolites and organic acids that facilitate mineral transport across intestinal cells. Likewise, fermentation-induced pH reduction and protein modification can enhance calcium bioavailability by maintaining minerals in a soluble form during intestinal transit (34, 35). The modest yet significant increase in zinc bioavailability may be related to improved mineral stability and reduced competition from inhibitory dietary components following fermentation and digestion. Although bioavailable mineral levels increased markedly in the buttermilk-treated samples, bioavailability represents only the fraction of bio accessible minerals taken up by Caco-2 cells under low- phytate conditions and does not imply complete or proportional whole- body human absorption. This reflects improved mineral solubility and enhanced epithelial uptake under optimized experimental conditions, rather than exaggerated absorption. This distinction indicates the inherent limitations of *in vitro* cell models, where nutrient transporters, competitive ion interactions, and cellular regulation determine the extent of uptake from the bioaccessible pool (36). The functional qualities of adjuvant (*anupāna*), in accordance with Ayurvedic principles, can be attributed to the enhanced bioaccessibility and bioavailability of iron, calcium, and zinc. *Āyurveda* defines *Anupāna* as a substance that is purposefully served in conjunction with diet or medication to improve the utilization of the principal substance. *Anupāna*, like buttermilk, helps to break down food mass, soften, moisten, digest, and facilitate absorption in the context of diet (37). Hence this study demonstrates that buttermilk, improves food breakdown, exposes more surface area to enzymes, softens food, helps with digestion, and makes it easier for nutrients to traverse the intestinal epithelial cells, promoting effective circulation.

Within the *Asta Āhāra Vidhiviseṣāyatanāni* (principles of dietetics in Ayurveda) framework, the synergistic or antagonistic effect of *saṃyoga* (combined effect of two or more things) (38), particularly in the case of buttermilk and greengram, plays a significant role in influencing the bioavailability of essential micronutrients. This association may enhance mineral solubility and support gut health by lowering anti- nutritional factors like phytate in green gram. The study’s limitations include the constrained generalizability in in-vitro experiments, which may not fully capture real-world scenarios or populations, the simplification of the human intestinal environment with the use of Caco-2 cells, and the likelihood of overlooking temporal variations in nutrient bioavailability owing to single-timepoint measurements and probable constraints in ICP-MS analysis.

## 5 Conclusion

The interaction between buttermilk, acting as a dephytinizer by its inherent phytase enzymatic activity, and green gram gave rise to enhanced bioaccessibility and led to improved nutrient availability. This study reinforces Ayurvedic principles by demonstrating the significant enhancing effect of the specific *anupāna,* buttermilk on the bioaccessibility and bioavailability of essential micronutrients like iron, calcium, and zinc in green gram. The incorporation of buttermilk into the diet emerges as an effective and economical strategy to transform a nutritionally suboptimal diet into a highly nutritious one. Embracing Ayurvedic dietetics could serve as a sustainable approach to address malnutrition and related diseases. These principles should be communicated at the individual level through health education and counseling by professionals, and at the national level through complementary nutrition policies to ensure both qualitative and quantitative nutritional sufficiency.

## Data Availability

The original contributions presented in the study are included in the article/supplementary material, further inquiries can be directed to the corresponding author.
